# Inferring signalling networks from images

**DOI:** 10.1111/jmi.12062

**Published:** 2013-07-11

**Authors:** L Evans, H Sailem, P Pascual Vargas, C Bakal

**Affiliations:** Chester Beatty Laboratories, Division of Cancer Biology, Institute of Cancer Research237 Fulham Road, London, UK, SW3 6JB

**Keywords:** Heterogeneity, image analysis, morphological signatures, RNAi, signalling networks

## Abstract

The mapping of signalling networks is one of biology’s most important goals. However, given their size, complexity and dynamic nature, obtaining comprehensive descriptions of these networks has proven extremely challenging. A fast and cost-effective means to infer connectivity between genes on a systems-level is by quantifying the similarity between high-dimensional cellular phenotypes following systematic gene depletion. This review describes the methodology used to map signalling networks using data generated in the context of RNAi screens.

## Introduction

A central challenge in biology and medicine is to comprehensively map cellular signalling networks. Signalling networks receive input from the environment and regulate fundamental cellular behaviours such as proliferation, metabolism, morphogenesis and death. Ideally, network maps should be quantitative descriptions of how different components physically interact, and be predictive of how information flows through the network in response to stimuli. Although early studies of signal transduction derived descriptions of hierarchical and linear pathways consisting of a limited number of proteins (e.g. in the order of 10–20 proteins), it is clear that these representations are not reflective of *in vivo* network architecture and dynamics. Specifically, signalling networks involve 1000s of different components, signalling pathways are highly interconnected, proteins act as part of large complexes, information propagation occurs via both linear and nonlinear ways (e.g. by feedback and oscillations) and these networks are dynamical in nature (Bork & Serrano, 2005; Ferrell *et al*., 2011; Vidal *et al*., 2011).

Despite broad conceptual advances in our understanding of cell signalling, most signalling networks remain unexplored. It is sobering to consider that it has required a significant effort over the last decade to generate a comprehensive genetic interaction map for the single cell organism *Saccharomyces cerevisiae* growing in one type of media (Costanzo *et al*., 2010), however, networks are likely to differ tremendously based on cell-type and environmental conditions. Large-scale efforts to map protein–protein interactions (PPIs) cover only small portions of network space (Rual *et al*., 2005; Guruharsha *et al*., 2011). Thus if we are to map networks in many different cell types, from different genetic backgrounds, across diverse conditions, fast and cost-effective methods are required to infer connectivity between components.

**Figure 1:**
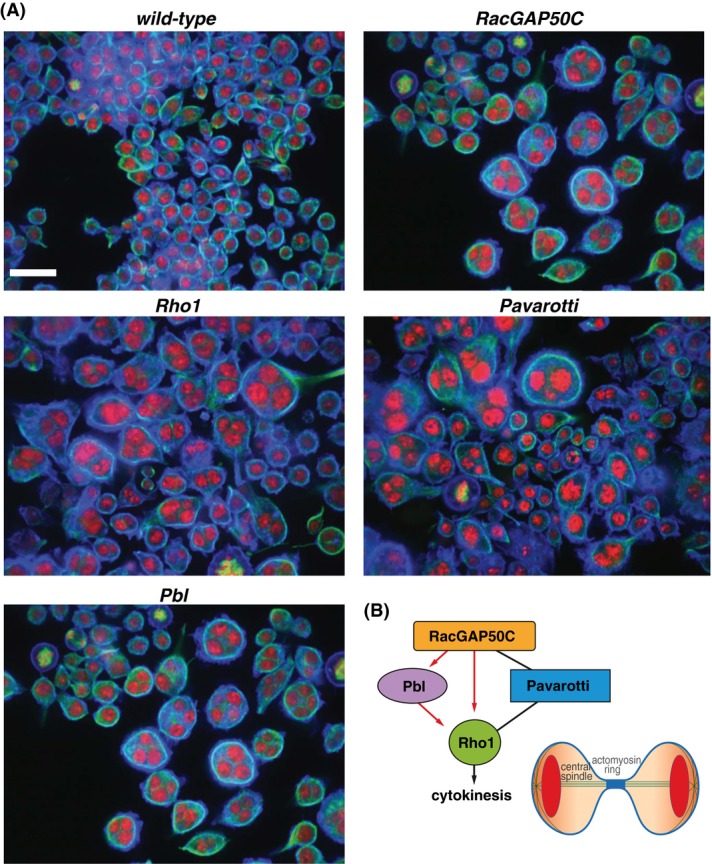
Depleting different members of the same signalling complex leads to similar cellular phenotypes. (A) Kc167 *Drosophila* cells treated with dsRNA targetting *Rho1*, *Pbl*, *RacGAP50C* or *Pavarotti*, have similar cellular phenotypes. Cells were treated with RNAi and then fixed, stained with DAPI (red), phalloidin (blue), and anti-alpha tubulin antibody (green), and then imaged using an Opera QEHS microscope (PerkinElmer). The appearance of large, multinucleated, round cells suggests cytokinesis, but not mitosis, has failed during cell division. Scale bars are equal to 20 μm. (B) Rho1, Pbl, RacGAP50C and Pavarotti act as part of the same signalling complex during cytokinesis. All proteins localize to the presumptive cleavage furrow and promote actomyosin contractile ring assembly. Red arrows indicate there is evidence for active regulation of one protein by another, such activation/inhibition or control of localization.

Genetics has always had an important role to play in describing signalling systems. The power of genetics derives from the fact that depletion of genes encoding members of the same signalling pathway or complex leads to similar cell and organism phenotypes. Figure 1 shows a simple example, where depletion by RNA interference (RNAi) of *RacGAP50C*, *Rho1, Pbl* or *Pavarotti* in *Drosophila* Kc167 cells leads to strikingly similar phenotypes – multinucleate cells that have successfully progressed through mitosis (as they show normal nuclear morphology), but failed in the late stages of cytokinesis. Thus, based on the shapes of the nuclei and the cell itself following gene depletion, we can infer that these genes regulate the same function (assembly of the contractile ring during cytokinesis), potentially make physical interactions, and may regulate the spatiotemporal activity of each other. All of these inferences regarding *RacGAP50C*, *Rho1*, *Pbl* and *Pavarotti* have in fact been validated using both forward genetic and biochemical methods in a number of different organisms (Fededa & Gerlich, 2012). Even this simple example shows how deep insights into signalling networks can be gained from a set of static images of mutant phenotypes.

Classically, assessment of complex cellular phenotypes such as cell shape following genetic perturbation has been performed qualitatively and in relatively low throughput. For example, many cell biology studies still involve human interpretation of images derived from a small number of experiments, resulting in limited descriptions of signalling pathways. However, recently developed, automated high-throughput cellular imaging methodologies can now be used to quantitatively describe signalling networks following unbiased, large-scale, systematic gene depletion by RNAi. It is these methodologies that will be the subject of this review, with particular focus on the initial extraction of raw data from images and the methods used to map networks following this extraction.

## Experimental set-up

Generating a dataset of images following systematic gene depletion for the intent of describing signalling networks involves the same methods as performing any image-based RNAi screen. These protocols have been extensively reviewed elsewhere and will only be discussed briefly here (Mohr *et al*., 2010). In high-throughput experiments, each RNAi is prealiquoted in a particular well of a multiwell plate (96, 384 or 1536 wells per plate), and cells are cultured in each well for a period of ∼2–6 days. Hundreds to thousands of cells can be grown in each well. Given that an experiment might involve imaging of 1000–1 000 000 individual wells, this is performed using high-throughput microscopes. The inclusion of not only appropriate controls, as well as both technical and experimental replicates is critical to the analysis and interpretation of these experiments. Technical replicates are required to account for the high degree of variability observed between the effects of the same RNAi in the same plate, and between duplicate plates that are prepared on the same day. If experiments are performed many weeks or months apart, technical replicates are also important to take ‘batch effects’ into account. Experimental replicates, where different siRNA, shRNA or dsRNAs are used to target the same gene, are required to account for off-target effects (that generate false positives) and ineffective knockdowns (that generate false negatives). Although experimental replicates are essential to the experimental design, their inclusion also leads to specific challenges in describing cellular phenotypes and thus network mapping, as the effects of different RNAis can vary tremendously even though they target the same gene. The number of genes that are analysed in screens has a direct effect on the granularity of the outcome, or the number of unique interactions that will be identified; published screens have ranged in size from ∼250–25 000 genes.

It is important to differentiate attempts to map signalling networks using high-throughput genetics from conventional RNAi screens where the goal is to identify a limited number of genes that contribute to a phenotype of interest. In conventional RNAi screens different scoring metrics are employed to differentiate ‘hits’ from ‘nonhits’ and great emphasis is placed on lowering false positive rates, potentially at the risk of increasing false negatives. From the perspective of the data analysis, this is a ‘finding a needle in haystack’ type of problem. However, when mapping signalling networks, the challenge is to assign a phenotypic ‘signature’ to all genes and determine similarities/differences in these signatures – or a ‘hay vs. hay’ analysis.

## The phenotype

An important consideration in building network maps using imaging data is that phenotypes should have high dimensionality to identify as many different phenotypes as possible in the assay. By increasing the number of phenotypes, this increases the probability that the interactions assigned between genes based on the similarity of their phenotype following knockdown will be indicative of a genuine biochemical interaction that occurs *in vivo*. An analogy would be attempting to classify different dog breeds from a mixed population; if only differences in height from the mean is used as a readout, the small dog and large dog group would still contain a mix of breeds. However, if additional information, such as coat length, colour, ear shape, etc. were used, accurate classification is possible. However, many conventional assays used in molecular cell biology do not satisfy this condition of high dimensionality. For example, screens that measure cellular viability (Boutros *et al*., 2004) will not provide insight into network structure because only two different groups will be identified; genes whose depletion increases viability, and those who decrease it. Although some of the genes/proteins in each of these groups are likely to make interactions *in vivo*, there are a number of different means by which RNAi could affect viability, thus assigning interactions using only two groups will result in a number of false positive interactions.

**Figure 2:**
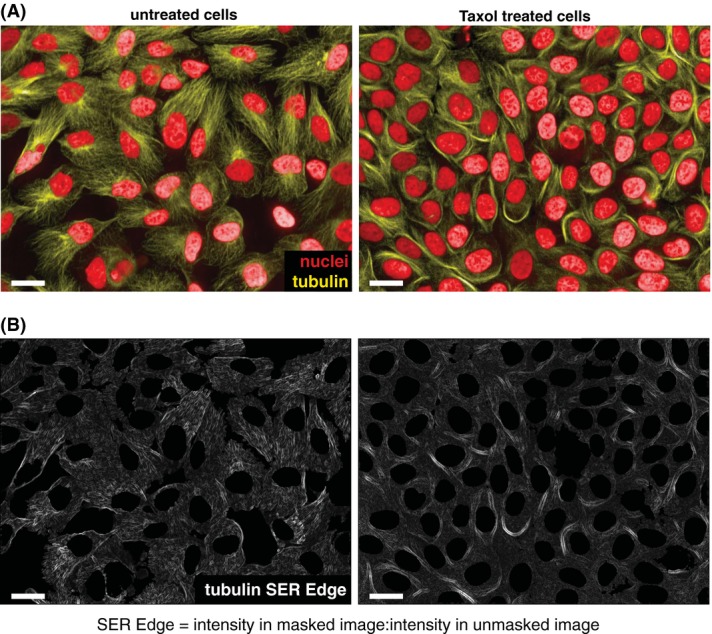
Texture features are useful descriptors of cellular phenotypes. (A) Untreated (left panels) or Taxol treated MCF-10A cells (right panels) were stained with DAPI (red) and antialpha tubulin antibody (yellow). Cells were imaged on an Opera QEHS microscope. ‘SER Edge’ filtered image generated in Columbus (PerkinElmer). (B) The SER Edge feature is calculated on a single cell basis as the pixel intensity within a single object (cell segment) averaged over the corresponding object. Scales bar are equal to 20 μm.

Cellular morphology has proved to be an excellent high-dimensional readout for network mapping studies (Bakal *et al*., 2007; Fuchs *et al*., 2010). In large part this is because of the fact that hundreds of different parameters describing cell shape can be extracted from single cells that have been labelled using any reagent (e.g. expression of EGFP, F-actin staining by phalloidin) that allows segmentation of cellular boundaries. Many different freely available (Bakal *et al*., 2007, Pau *et al*., 2010; Kamentsky *et al*., 2011;) or commercial packages are now available to extract such features from image datasets in a highly automated fashion. Furthermore, because many diverse cellular processes affect morphology (i.e. proliferation, growth, migration, differentiation and apoptosis), cell shape can be used to describe networks regulating these behaviours either in isolation or in parallel with other assays. An obvious caveat to the use of cell shape as a phenotypic readout is that genes whose depletion does not affect shape will not be included in network maps. In addition, even when scoring a complex phenotype such as shape, dysregulation of different processes can potentially lead to similar cell morphologies. Devising and measuring specific features that describe the effects of gene depletion, but are not necessarily affected by the overall cellular geometry and hence are able to differentiate between two qualitatively similar shapes can possibly address this issue. One excellent example are ‘texture features”’ (Haralick *et al*., 1973) where images are first filtered using particular patterns, and pixel intensities of the filtered image are calculated (Fig. 2). Taken together, from one single cell labelled with a single fluorescent reporter, 100–250 features can be extracted that describe cell geometry, label/reporter intensity, and patterns of texture. As many as ∼600 single cell features can be generated when multiple reporters (for example, DAPI is used to label nuclei and quantify nuclear shape) are used in parallel. From each well/experiment many thousands of single cells can be imaged. Thus the datasets that can be assembled can comprise millions to even trillions of individual data points. In fact, the bottleneck in these experiments is not in data acquisition, which is both fast and relatively cost-effective, but in data analysis.

We have recently implemented a multiplexed approach to generate high-dimensional signatures following different gene depletions by combining averaged single-dimensional readouts such as cell viability and levels of reactive oxygen species with basic measurements of cell morphology – all of which were quantified in different experiments (Garcia *et al*., 2012). Although these ‘virtual high-dimensional signatures’ makes single cell analysis impossible because the same cells are not being analysed in different assays, they allow the inference signalling networks in a similar manner as performing a single high-content assay.

## Dimensionality reduction

Just as extracting a high number of features is a potentially powerful means to make subtle discriminations between cellular phenotypes, many of these features are likely to be uninformative (don’t vary), noisy (vary in nonmeaningful ways), and be highly correlated. For example, in Figure 1(A) actin intensity is not a useful feature to differentiate phenotypes. Returning to the dog classification analogy, leg number is likely to be an uninformative variable, whereas eye colour is a noisy variable. In addition, the use of many features is not recommended when using most machine learning and statistical analysis methods because of ‘the curse of dimensionality’, where the analysis power decreases with the number of features measured. The curse of dimensionality is especially problematic in image analysis, where the number of phenotypic features that can be measured from a single image continues to grow as both microscopy hardware and software improves. An important challenge is thus to transform a high-dimensional data set derived from images to a low-dimensional informative dataset with minimal information loss. Dimensionality reduction is not unique to imaging studies as similar challenges are faced in the reduction of high-dimensional datasets such as those generated by expression profiling, where the goal is to identify a set of genes whose expression levels can be used to best separate different samples.

Dimensionality reduction can be performed in either a supervised or unsupervised fashion. In supervised methods, a set of mutant phenotypes must be first identified *a priori*, and then algorithms are used that select a subset of features that can discriminate these phenotypes from wild-type cells. Unsupervised approaches do not require any prior knowledge about the data, and attempt to identify the main trends of variability in the data by representing these trends in low-dimensional space. Principal component analysis (PCA) is a popular method of unsupervised dimensionality reduction. However, a caveat to using unsupervised methods like PCA is that it weighs features based on their variance rather than their relevance to the phenotypes of interest.

## Assigning signatures to phenotypes

We have previously made use of supervised approaches where we used machine learning algorithms to find combinations of features, or classifiers, that best explain our data (Bakal *et al*., 2007). Specifically we preselected seven different ‘reference’ or ‘exemplar’ phenotypes that human observers considered qualitatively different from wild-type cells, as well as each other, and then generated neural networks that identified combinations of features which could quantitatively distinguish these phenotypes. Subsequently, the entire dataset was then scored using these features. Different phenotypes were thus classified as to how similar/different they were to the reference phenotypes, which allowed us to quantify even subtle differences between populations. That human observers must first isolate phenotypes of choice is a considerable disadvantage to the use of supervised methods, especially if it is unclear what such phenotypes might be before the images are acquired. In the context of large screens where millions of images are acquired, it is not feasible to examine the entire dataset. Furthermore, there is a danger that the use of such methods fails to fully classify the wide range of phenotypes that are actually present in the data, and that the captured variance is heavily biased. However, we have recently described the use of automated phenotypic detection algorithms that can be implemented to identify reference phenotypes from a dataset of choice (Yin *et al*., 2008). Once the relevant features have been identified, any cell can be assigned a ‘phenotypic signature’, or ‘Quantitative Morphological Signature’ that describes its phenotype in a multidimensional manner.

## The challenge of phenotypic heterogeneity

A nontrivial aspect to the analysis of high-content screens and the inference of signalling networks is how to derive a single phenotypic signature of highly heterogeneous cell populations. The most straightforward, and frequently used, method to calculate a single signature for a gene is to calculate the average phenotypic signature of cells from all populations where RNAi has depleted the gene, and this has been frequently used in the past. However, it is clear that even wild-type/untreated cells can exhibit high levels of phenotypic heterogeneity that may be driven by stochastic fluctuations in transcription, protein levels, signalling activity and cytoskeletal polymerisation (Mitchison & Kirschner, 1984; Spencer *et al*., 2009; Altschuler & Wu, 2010; Balazsi *et al*., 2011). Moreover, in some cell types the spatial distribution of a cell within a colony can have effects on cellular behaviour (Snijder *et al*., 2009). In RNAi-treated cells, heterogeneity can be further exacerbated by factors such as incomplete penetrance and pleiotropic effects of RNAi. Notably, different RNAis (e.g. different siRNA from the same and/or different vendors) can also often lead to different phenotypes, even though they target the same gene (Collinet *et al*., 2010). Therefore, the heterogeneity of: (i) the wild-type population, (ii) the population in each RNAi experiment (e.g. single wells) and (iii) different populations from technical (repeat wells) and experimental (different RNAi reagents targeting the same gene) repeats, must be accounted for to calculate a single phenotypic signature that describes depletion of a gene. Only then can different gene-specific signatures be compared. Recent high-content screens have implemented novel methods to determine the most common phenotype amongst experimental replicates (Collinet *et al*., 2010), and to consider the heterogeneity of different populations when measuring the effects of gene depletion (Snijder *et al*., 2012). In the latter case, Snijder *et al*. generated models that assume different cells will have different phenotypes following depletion of the same gene by RNAi based on their position in multicellular colonies. Accounting for positional effects essentially ‘normalizes’ some of the phenotypic heterogeneity that is commonly observed following RNAi (Snijder *et al*., 2012). That population heterogeneity can be accounted for in this manner is one significant advantage to generating single cell high-content data.

## Grouping quantitative signatures and inferring networks

Once a multidimensional gene specific signature has been calculated, measuring the similarity between signatures, and inferring connectivity, can be straightforward. A common method to infer interactions between genes is to use clustering methods that group gene signatures into ‘phenoclusters’ based on a given similarity metric, such as Euclidean Distance or Pearson correlation. In a screen for genes regulating cell morphology, we have shown that co-membership in a phenocluster strongly suggests that two genes encode members of the same signalling pathway/complex (Bakal *et al*., 2007). In this case each phenocluster can also be considered a ‘local network’ of proteins that act in distinct spatiotemporal manners to regulate a specific morphological process (e.g. protrusion, lammellipodium formation). The notion that clustering of mutant phenotypes is indicative of two proteins acting as part of the same signalling complex is further supported by yeast and worm studies which have performed both image analysis of mutant phenotypes in parallel with large-scale quantification of PPI (Gunsalus *et al*., 2005; Gavin *et al*., 2006). That protein function, subcellular localization, and PPI can be inferred from static images underscores the power of high-throughout and high-content genetics.

One caveat to the use of similarity-based classification schemes such as hierarchical clustering is that phenoclusters are essentially complete graphs where each gene/protein is connected to all other in the cluster, and all clusters are ultimately connected to each other. Unfortunately, although clustering recapitulates the idea that many proteins that regulate a common function often act as part of large signalling complexes, it cannot be used to describe finer aspects of signalling network architecture and dynamics. For example, it is unlikely that all proteins assigned to a particular phenocluster physically interact. Moreover, using clustering it is essentially impossible to identify situations where a particular protein may act as an upstream regulator of another.

To overcome these problems, two methods have been successfully implemented to use phenotypic data generated by screens to infer more physiological aspects of signalling network architecture and dynamics. The first is to integrate phenotypic data with other data sources, such as coexpression or PPI data (Gunsalus *et al*., 2005; Fuchs *et al*., 2010). In the simple case, when phenotypic signatures of two genes/proteins are highly correlated, a connection between them is inferred only if they have previously been shown to physically interact and/or be co-expressed. In more complex integration procedures, all data sources are used in models that consider the probability of each type of interaction or correlation and then generate a connection based on this interaction. In the latter case, if the correlation between phenotypic signatures is high, a connection can potentially be assigned even in the absence of any other supporting data, whereas in simple integration schemes, one data source essentially validates the other. Data integration methods are particularly useful when attempting to map very large networks, such as those generated by genome-wide screens. Using prior knowledge is an alternative to data integration that can also be used to infer signalling network architecture. For example, we have previously modelled a signalling network comprised of Rho GTPase Activating Proteins (RhoGAPs) from image-based data by assuming that RhoGAPs act upstream of their target Rho-family GTPases. Using image-based data we could determine previously unknown enzyme-substrate interactions and make inferences regarding signalling dynamics (Nir *et al*., 2010). However, the obvious limitation to this method is that it requires prior knowledge that may not exist for many other types of proteins, and the final network will be constrained by the assumptions used to construct it. For example, we could not incorporate into our model the fact that RhoGTPases can also potentially act upstream of different RhoGAPs. Devising new means of modelling signalling networks from imaging data is currently an intensive area of research.

## The future

In the past many groups, including our own, have been focused on development of the methods themselves, but the challenge now is to use these methods across many different cell lines and/or conditions in a genome-wide fashion to gain insight into how genetic backgrounds and environmental conditions affect network architecture. Furthermore, it is essential that new computational methods be developed which can be used to model aspects of signalling dynamics such as information flow, oscillatory behaviour and feedback. Finally, as imaging instrumentation becomes faster, and data storage ever cheaper, the task of analysing extremely large datasets in an efficient manner will require new solutions. In particular, solving the curse of dimensionality with regards to high-content data will require extensive collaborations between cell biologists and mathematicians.
